# Transcutaneous auricular vagus nerve stimulation enhances short-latency afferent inhibition via central cholinergic system activation

**DOI:** 10.1038/s41598-024-61958-8

**Published:** 2024-05-16

**Authors:** Takayuki Horinouchi, Tomohisa Nezu, Kazuya Saita, Shota Date, Hiroshi Kurumadani, Hirofumi Maruyama, Hikari Kirimoto

**Affiliations:** 1 Department of Sensorimotor Neuroscience, Graduate School of Biomedical and Health Sciences, Hiroshima University, 1-2-3 Kasumi, Minami-ku, Hiroshima, 734-8553 Japan; 2 Department of Clinical Neuroscience and Therapeutics, Graduate School of Biomedical and Health Sciences, Hiroshima University, 1-2-3 Kasumi, Minami-ku, Hiroshima, 734-8553 Japan; 3 Japan Society for the Promotion of Science, Tokyo, Japan; 4 Department of Psychosocial Rehabilitation, Graduate School of Biomedical and Health Sciences, Hiroshima University, Hiroshima, Japan; 5 Department of Analysis and Control of Upper Extremity Function, Graduate School of Biomedical and Health Sciences, Hiroshima University, Hiroshima, Japan

**Keywords:** Non-invasive brain stimulation, Transcutaneous auricular vagus nerve stimulation, Short-latency afferent inhibition, Cholinergic neural circuit, Neuroscience, Neurology

## Abstract

The present study examined the effects of transcutaneous auricular vagus nerve stimulation (taVNS) on short-latency afferent inhibition (SAI), as indirect biomarker of cholinergic system activation. 24 healthy adults underwent intermittent taVNS (30 s on/30 s off, 30 min) or continuous taVNS at a frequency of 25 Hz (15 min) along with earlobe temporary stimulation (15 min or 30 min) were performed in random order. The efficiency with which the motor evoked potential from the abductor pollicis brevis muscle by transcranial magnetic stimulation was attenuated by the preceding median nerve conditioning stimulus was compared before taVNS, immediately after taVNS, and 15 min after taVNS. Continuous taVNS significantly increased SAI at 15 min post-stimulation compared to baseline. A positive correlation (Pearson coefficient = 0.563, *p* = 0.004) was observed between baseline SAI and changes after continuous taVNS. These results suggest that 15 min of continuous taVNS increases the activity of the cholinergic nervous system, as evidenced by the increase in SAI. In particular, the increase after taVNS was more pronounced in those with lower initial SAI. This study provides fundamental insight into the clinical potential of taVNS for cholinergic dysfunction.

## Introduction

Noradrenergic and cholinergic systems exhibit distinct projection patterns within the brain. The noradrenergic locus coeruleus (LC) disperses extensive projections throughout the entire cortex^1^, while the cholinergic nucleus basalis of Meynert projects in a more selective manner to discrete regions across the cerebral cortex^1,2^. As a result, there are significant disparities in the impacts of these noradrenergic^3^ and cholinergic systems^4^ on cognition and behavior. Nevertheless, recent studies have revealed that both systems share numerous commonalities and collaborate to facilitate adaptive cognition, encompassing processes such as learning, memory, attention, and decision-making^5–7^.

Over the past decade, studies on transcutaneous auricular vagus nerve stimulation (taVNS) have consistently demonstrated the utility of the concha and tragus, regions innervated by the auricular branch of the human vagus nerve, as valuable gateways for non-invasive brain stimulation^8–11^. This emerging technique has garnered considerable attention for its potential in treating central nervous disorders, as well as for enhancing cognitive functions in healthy subjects^12–16^. However, despite these promising outcomes, the precise mechanisms underlying these effects have not been completely elucidated. Functional magnetic resonance imaging (fMRI) studies have suggested that taVNS may function by activating the nucleus of the solitary tract (NTS)^17^ and the locus coeruleus-norepinephrine (LC-NE) system^18,19^, which receive projections from the NTS. Additionally, it has been suggested that physiological markers, such as the P300 amplitude of event-related potentials^20^, salivary alpha-amylase levels^20^, and pupil dilation^21^, may serve as supportive biomarkers of noradrenergic-related processes. Similarly, animal studies have provided insight into the neural connections from the vagus nerve to the cholinergic basal forebrain (BF) via the NTS^22^, observing an association between the cholinergic neural system activation and enhanced reinforcement learning using implanted electrode-based Vagus Nerve Stimulation (VNS)^23^, as well as the alleviation of sevoflurane-induced cognitive dysfunction through taVNS^24^. Nonetheless, the impact of taVNS on the cholinergic neural system in humans remains an area that requires further exploration.

**Figure 1:**
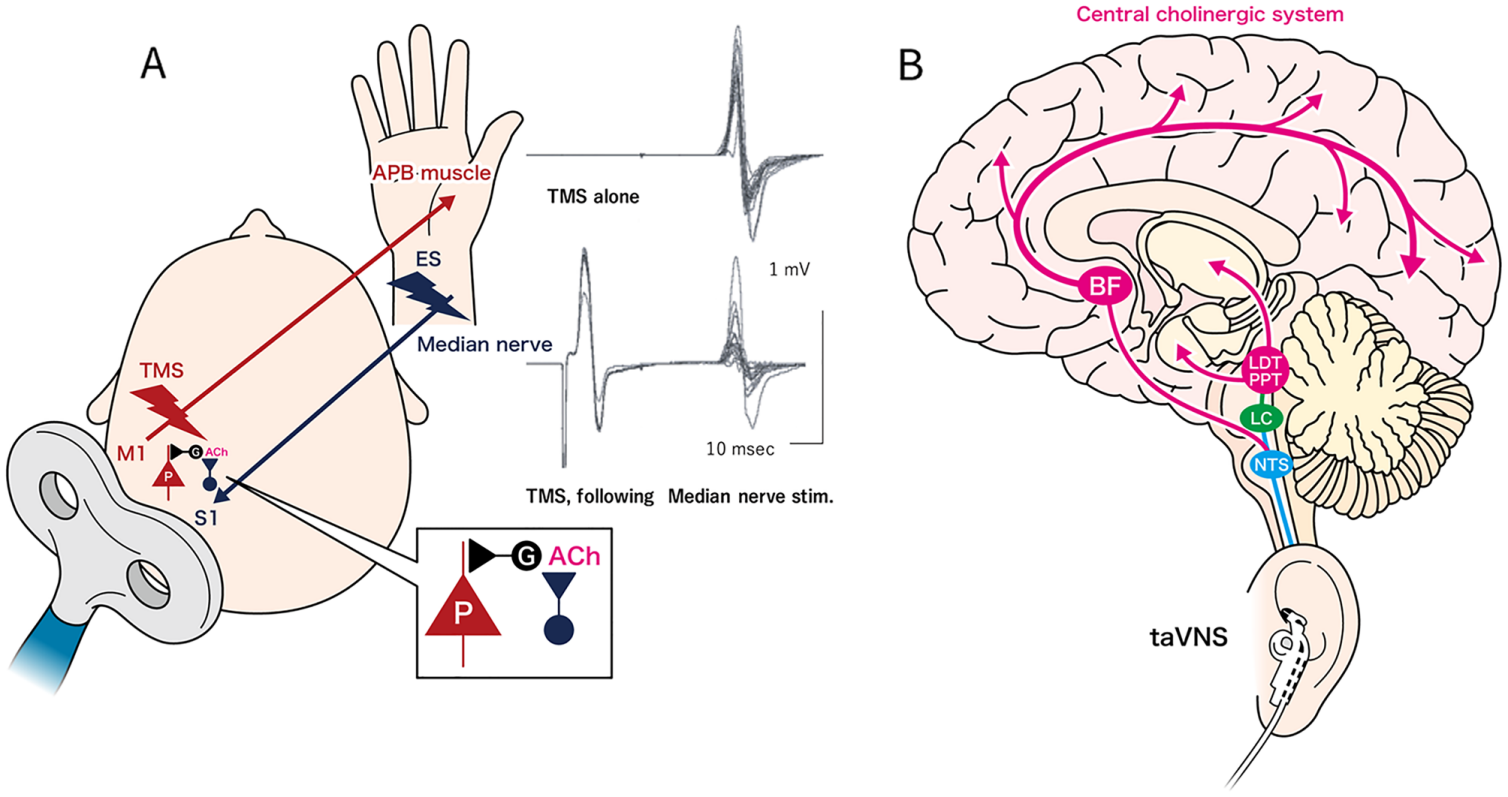
This method assesses motor cortex excitability by measuring MEPs in response to a peripheral nerve electrical stimulus. Somatosensory afferent input activates cholinergic neurons, reducing MEP amplitudes and inhibiting pyramidal cell (P) transmission from S1 to M1 via GABAergic interneurons (G). This phenomenon is called short-interval afferent inhibition (SAI) and has rendered SAI a dependable neurophysiological indicator of cortical cholinergic activity (**a**). It has been demonstrated in animal experiments that taVNS activates cholinergic neural circuits, but it is not clearly understood in humans. This study revealed an enhancement of SAI following taVNS, suggesting activation of cholinergic neural circuits (**b**). MEP, motor evoked potentials; TMS, transcranial magnetic stimulation; APB, abductor pollicis brevis; M1, primary motor area; S1, somatosensory motor area; taVNS, transcutaneous auricular vagus nerve stimulation; NTS, nucleus tractus solitarie; LC, locus coeruleus; PPT, pedunculopontine tegmental nucleus; LDT, laterodorsal tegmental nucleus; BF, basal forebrain.

The cholinergic system can be examined in vivo using short-latency afferent inhibition (SAI), which is a transcranial magnetic stimulation (TMS) paradigm^25,26^. This non-invasive method assesses the excitability of the motor cortex (M1) by measuring the amplitudes of motor-evoked potentials (MEPs) in response to a conditioning sensory afferent electrical stimulus applied to a peripheral mixed nerve. If the interval between the peripheral nerve conditioning stimulus and the TMS pulse over M1 is slightly longer than the N20 latency of the somatosensory evoked potentials (SEPs), there is a decrease in the amplitude of MEPs^25^. This reduction results from the activation of cholinergic neurons, which subsequently excite GABAergic interneurons, inhibiting pyramidal cells during the transmission of sensory signals from the primary somatosensory cortex (S1) to M1^27–29^ (Fig. 1). Under normal conditions, scopolamine, a muscarinic antagonist, is known to abolish or diminish SAI^30^, while in Alzheimer's disease the acetylcholinesterase inhibitor rivastigmine has been observed to enhance SAI^31^. Further, SAI has provided valuable insights into various cholinergic neural circuit disorders that impact cognition and motor function, including Alzheimer's disease^31,32^, idiopathic normal‑pressure hydrocephalus^33^, Parkinson’s disease^34,35^, and dystonia^30,36^. These insights have rendered SAI a dependable neurophysiological indicator of cortical cholinergic activity, well-suited for examining the impacts of interventions aimed at addressing cortical cholinergic dysfunction^29^.

In previous studies evaluating the effect of taVNS on SAI, Midden et al.^37^ have reported that 100 Hz continuous taVNS increased short-latency intracortical inhibition (SICI)^38^ with no changes in SAI. Meanwhile, there is consensus that cholinergic interneurons mediating SAI and GABAergic interneurons responsible for SICI interact bidirectionally in the cortex^26,29,39^, and interestingly, excitability of cholinergic interneurons can be increased by strong inhibition from GABAergic interneurons. Moreover, changes in peripheral sensory input intensity or interventions can result in increased SAI without changes in SICI or decreased SAI with increased SICI^28,40,41^. Regarding the VNS, it has been postulated that the relationship between optimal stimulation intensity and VNS-dependent neural plasticity exhibits an inverted U-shaped curve^42^. More importantly, the frequency range endorsed by the U.S. Food and Drug Administration (FDA) for effective seizure control is 10–30 Hz^43^. Considering these previous studies and the FDA endorsement, we speculate that the utilization of 100 Hz taVNS may be the reason why no changes in SAI were found in the study by Midden et al. In order to test this speculation, this current study was carried out to investigate in detail the effect of 25 Hz taVNS, which has been used in a relatively large number of studies, on SAI. In addition, we compared the continuous and intermittent taVNS since no definitive conclusion has been reached regarding which setting is more effective.

Therefore, the aim of this study was to ascertain whether intermittent or continuous 25 Hz taVNS activate the cholinergic neural network and enhance SAI function in healthy individuals. This is an essential pilot study to establish foundational data for future clinical applications of taVNS, as this technique holds great promise for ameliorating symptoms and aiding the rehabilitation of central nervous system disorders characterized by impaired cholinergic neural networks.

## Results

### Effects of taVNS on unconditioned and conditioned MEPs and SAI

**Figure 2:**
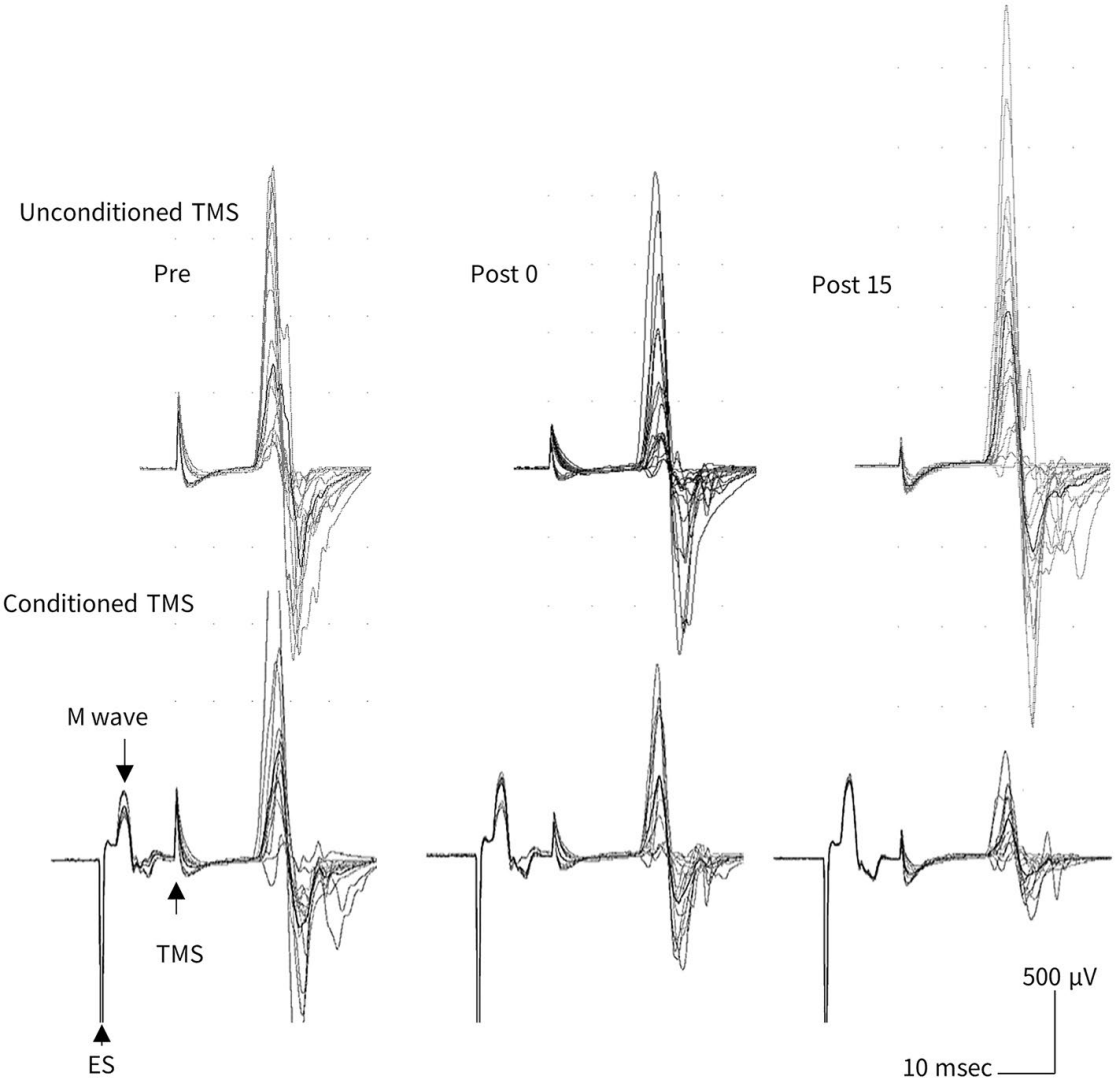
Representative MEP waveforms recorded from a subject before, immediately after, and 15 min after continuous taVNS.

**Figure 3:**
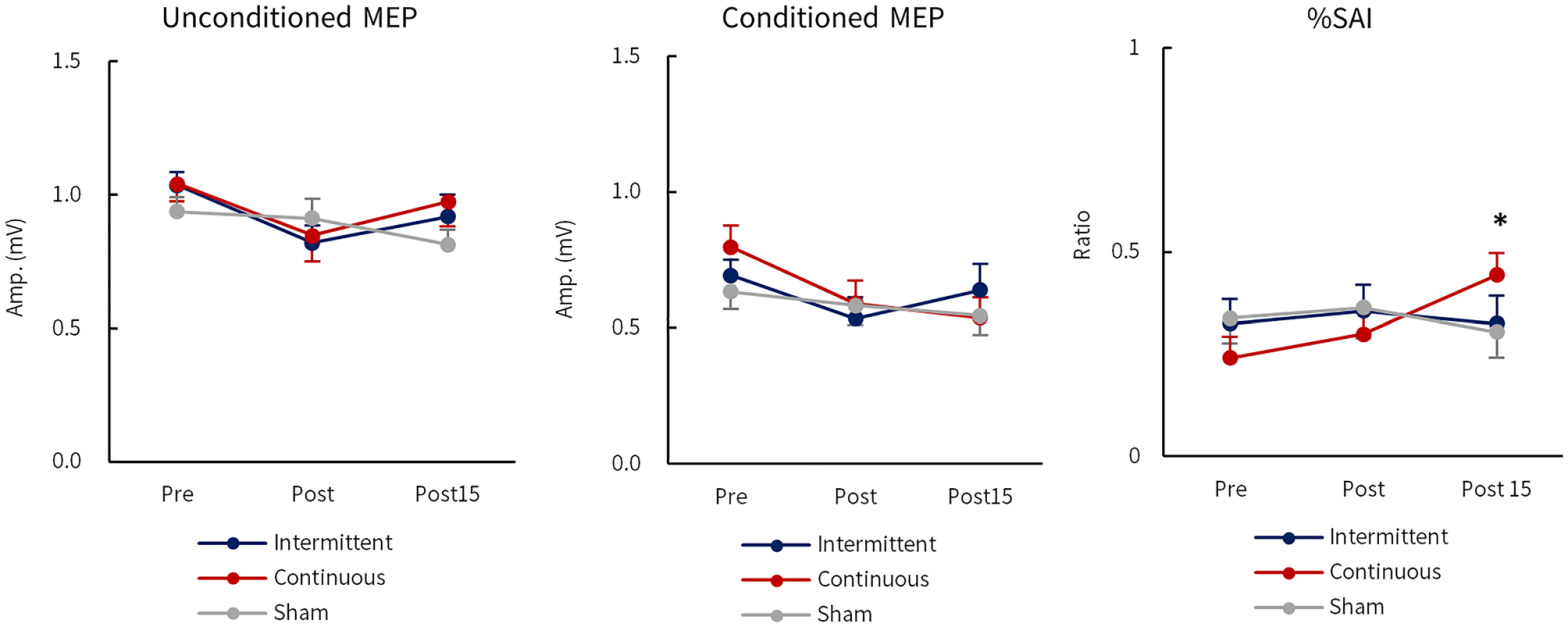
Serial changes in unconditioned (left) and conditioned (center) MEP amplitudes, and %SAI (right) before, immediately after, and 15 min after taVNS. No significant effect of taVNS was observed in the unconditioned and conditioned MEPs. Regarding %SAI, a two-way repeated measures ANOVA revealed an interaction effect between the taVNS condition and time. Post hoc analysis showed a significant difference between pre and 15 after continuous taVNS (*p < 0.05 vs. pre taVNS).

The sham taVNS, administered via the earlobe, tended to have higher tactile and pain thresholds compared to real taVNS (intermittent and continuous) delivered from the cymba conchae, although no statistically significant differences were observed between the two (Table 1). The amplitudes of unconditioned MEPs before administration of each taVNS stimulus condition were comparable: intermittent, 1.04 ± 0.05 mV; continuous, 1.04 ± 0.07 mV; sham, 0.94 ± 0.05 mV. Similarly, the amplitudes of the conditioned MEPs before each taVNS stimulus condition were comparable: intermittent, 0.69 ± 0.06 mV; continuous, 0.80 ± 0.08 mV; sham, 0.63 ± 0.06 mV. No significant main or interactive effects were observed on either unconditioned or conditioned MEP amplitudes before, immediately after, or 15 min after taVNS. The %SAI values under each taVNS stimulus condition were comparable: intermittent, 32 ± 0.06%; continuous, 24 ± 0.05%; sham, 34 ± 0.05%. Furthermore, the ICCs (3, 1) of %SAI before administration of the three taVNS conditions (intermittent vs. continuous, intermittent vs. sham, and continuous vs. sham) were 0.53, 0.61, and 0.64, respectively. Figure 2 shows MEP waveforms recorded from a representative subject before, immediately after, and 15 min after continuous taVNS. As shown in this figure, the decrease in MEP amplitude induced by conditioned TMS was observed only in the continuous taVNS condition, and no change occurred in the intermittent and sham taVNS conditions. Figure 3 shows serial changes in unconditioned (left) and conditioned (center) MEP amplitudes, and %SAI (right) before, immediately after, and 15 min after taVNS. No significant effect of taVNS was observed in the unconditioned and conditioned MEPs. Regarding %SAI, a two-way repeated measures ANOVA revealed an interaction between taVNS condition and time (*F*_4, 92_ = 3.315, *p* = 0.014, η^2^ = 0.126), but no significant main effects were observed for either taVNS condition or time. The %SAI significantly increased 15 min after continuous taVNS (*p* = 0.012, η^2^ = 0.314).

**Table 1:** Stimulation intensity, sensory/pain thresholds for taVNS, and stimulation intensity for TMS.

	taVNS		TMS	
	Stimulation intensity (mA)	Tactile threshold (mA)	Pain threshold (mA)	Stimulation intensity (%MSO)
Intermittent	1.30 ± 0.1	0.86 ± 0.07	1.77 ± 0.13	59.9 ± 2.32
Continuous	1.39 ± 0.11	0.92 ± 0.07	1.90 ± 0.16	62.6 ± 2.53
Sham	1.54 ± 0.12	1.02 ± 0.12	2.13 ± 0.18	60.5 ± 2.05

*MSO* maximum stimulus output.

### Relationship between baseline %SAI and Δ%SAI after taVNS

**Figure 4:**
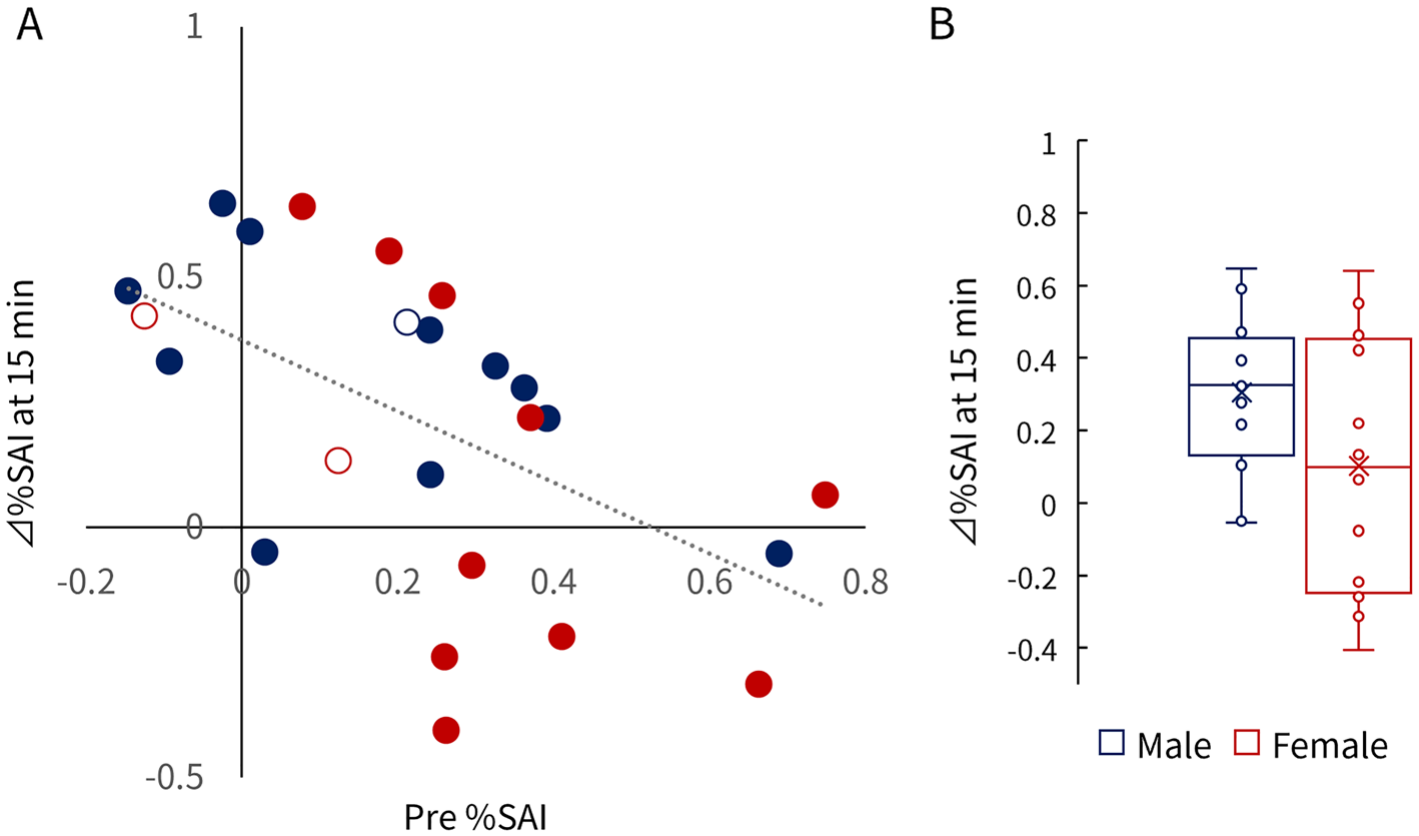
The relationship between baseline %SAI (Pre %SAI) and Δ %SAI 15 min after continuous taVNS (Δ %SAI at 15 min). Δ%SAI at 15 min demonstrates changes before taVNS and 15 min after continuous taVNS. (**A**); the Pearson correlation coefficient was 0.563 (*p* = 0.004). Blue represents males, red represents females, and open circles denote participants with Edinburgh Handedness Inventory scores < 70. Sex differences of Δ %SAI at 15 min after continuous taVNS (**B**).

The Pearson correlation coefficient for the relationship between baseline %SAI and Δ%SAI 15 min after continuous taVNS was 0.563 (*p* = 0.004) (Fig. 4A). There were six participants who were non-responders to taVNS, of which four were female. The Δ%SAI 15 min after taVNS tended to be larger in males than females, but the difference was not significant (Fig. 4B).

### Effects of taVNS on ECG and side effects

**Figure 5:**
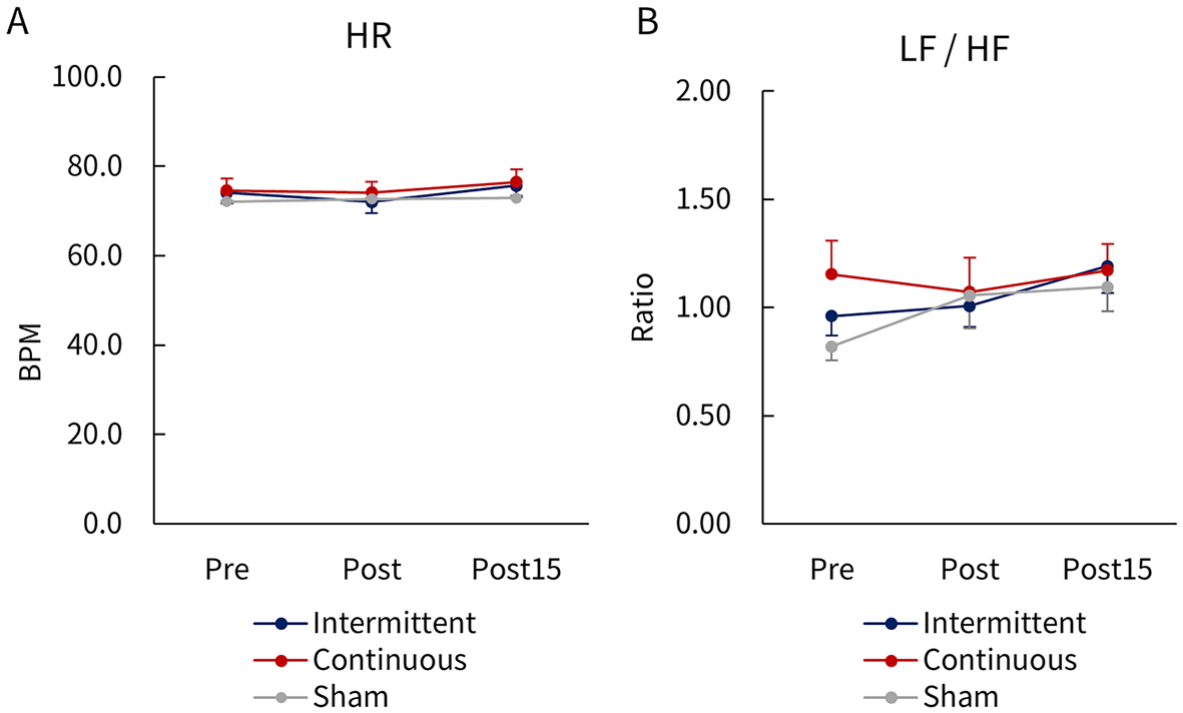
Serial changes in HR (**A**) and LF/HF (**B**) before, immediately after, and 15 min after taVNS. No significant main or interactive effects were observed on HR or LF/HF after taVNS.

No significant main effects or interactions were observed for HR (Fig. 5A) or the LF/HF ratio (Fig. 5B) after taVNS. Participants did not report physical changes or side effects during the whole study procedure.

## Discussion

In this study, we revealed that continuous taVNS, administered for 15 min at a frequency of 25 Hz, pulse width of 250 μs, and intensity set at the midpoint between tactile and pain thresholds, significantly boosted cholinergic system activity, as indicated by the enhanced SAI. Additionally, we observed a noteworthy correlation between %SAI and Δ%SAI after taVNS, demonstrating that individuals with lower initial SAI experienced more substantial increases in SAI following taVNS.

In our study, we found that the amplitudes of unconditioned and conditioned MEPs, as well as %SAI before taVNS, were quite similar among participants. Importantly, the intra-subject correlation coefficient of %SAI among different taVNS stimulation conditions was approximately 0.6, indicating a moderate reproducibility^40,41^. Therefore, we inferred that the enhanced SAI function observed following continuous taVNS was probably not due to issues with low intra-individual reproducibility that are typically associated with SAI assessments^40,41^. Instead, the enhanced SAI function may be linked to factors such as changes in attention, habituation, and fatigue resulting from repetitive measurements of participants. The lack of changes in HR and LF/HF suggested that the stimulus had no impact on the autonomic nervous system.

Regarding the neural pathways originating from peripheral sensory input that suppress corticospinal excitability and reduce MEP amplitudes, there is a growing consensus that cholinergic interneurons mediating SAI and the GABAergic interneurons responsible for short-latency intracortical inhibition (SICI)^38^ within the cortex interact bidirectionally^26,29,39^. Intriguingly, excitability occurs in cholinergic interneurons receiving strong inhibitory input from GABAergic interneurons^26,29,39^. Consequently, alterations in the intensity of peripheral sensory inputs or interventions can lead to changes such as increases in SAI without changes in SICI or decreases in SAI alongside increases in SICI^28,40,41^. Reports such that by van Midden et al.^37^ have observed increases in SICI with 100 Hz continuous taVNS without concurrent changes in SAI, which may reflect the complexity of the underlying mechanisms. Our study primarily focused on evaluating SAI, and while we did not assess SICI, 25 Hz continuous taVNS may be an optimal stimulus for modulating SAI while not affecting SICI.

In the context of the debate between intermittent and continuous taVNS, it is necessary to consider the total current load, calculated as current density (stimulation strength (A) / electrode size (cm^2^)) multiplied by total stimulation duration (pulse duration × number of pulses) in seconds (s) [C/cm^2^]. If the total current load significantly affects the modulation of SAI, continuous taVNS may yield intervention effects in half the time of intermittent taVNS with 30-s on/off cycles. Furthermore, if one stimulation type is more effective in inducing neural plasticity, it may lead to different intervention effects despite the total charge load being the same. Recent animal studies have suggested that continuous stimulation is essential for sustained intervention effects, observing that enhancements caused by duty-cycled VNS (30 s on/60 s off) rapidly dissipated^44^. In current clinical treatments, VNS is typically administered in a duty-cycle manner, such as 30 s on/60 s off^45^. This approach is chosen based on the assumption that duty-cycled stimulation carries a lower risk of nerve damage^46^. However, continuous taVNS has also been used in studies involving both healthy participants^12,20^ and patients with migraine^47^ and Parkinson’s disease^48^, and no adverse events have been reported. Our study is the first to demonstrate SAI enhancement in humans through taVNS and directly compare intermittent and continuous taVNS. While we cannot provide definitive conclusions due to lack of data for comparison, 25 Hz continuous stimulation at an intensity between the tactile and pain thresholds appeared suitable for regulating SAI.

Our taVNS stimulation intensity and sensory/pain thresholds were similar to those reported by Ventura-Bort et al.^20^, who demonstrated that taVNS can enhance the LC-NE system by increasing event-related potentials component P300 and salivary alpha-amylase. Sensory/pain threshold values converge toward a constant absolute value across environments, races, and genders, making them useful for comparing taVNS effects on various parameters. Nonetheless, individual ear sizes vary, as is illustrated by the availability of various sizes of commercial wireless earphones. Current taVNS attachments may struggle to maintain stable electrical stimulation without applying constant external pressure, such as by using elastic tape or similar methods.

VNS has been shown to activate the cholinergic basal forebrain (BF) in rats, inducing plastic changes in M1^22,49^ and enhancing motor learning^23^. Recent research in rats has also revealed that taVNS can ameliorate sevoflurane-induced cognitive impairment and reduce neuronal apoptosis, necroptosis, and microglial activation in the hippocampus via the BF^24^. Notably, animal studies have provided evidence of direct noradrenergic projections to the BF^50^ and human fMRI studies have observed structural connectivity between the cholinergic BF and noradrenergic LC^6^. While we cannot provide direct neurophysiological evidence, we speculate that taVNS, through one of the neural circuits mentioned above, activated the cholinergic BF, leading to the observed enhancement of SAI. Furthermore, the observed augmentation of SAI in our study did not occur immediately after taVNS, but rather 15 minutes after its completion, suggesting that mechanisms producing after-effects (offline effects) are involved post-stimulation, possibly related to synaptic plasticity following taVNS^51^. Fragons et al.^17^ in an fMRI study, reported a decrease in the observed BOLD signal enhancement in the subthalamic nucleus (STN) during taVNS, which was followed by an increase in LC activity after stimulation. Our results may similarly suggest that stimulation involving the auricular branch of the human vagus nerve (ABVN) requires time to activate the cholinergic system through multiple neural nuclei. Further in-depth research is needed to elucidate the neurophysiological mechanisms by which taVNS enhances SAI in the future.

As previously mentioned, past research has suggested that SAI can serve as a biomarker for cholinergic neuron function^26,52^, and the vagus nerve is known to project to the basal forebrain^22,23,48^. Our results indicate that individuals with lower SAI levels before stimulation tend to experience more significant intervention effects following vagus nerve stimulation. This suggested that there is a mutual relationship between SAI and vagus nerve activity, where alterations in SAI can influence the physiological impact of vagus nerve stimulation. Conversely, recent research has revealed a correlation between basal forebrain volume and SAI in healthy participants^53^. While this may initially appear contradictory—with larger volumes seemingly implying greater stimulation effects—several factors need to be considered. Firstly, the effectiveness of and sensitivity to VNS has a non-linear relationship as it is influenced by numerous factors and exhibits significant inter-individual variability. A larger basal forebrain might be associated with increased susceptibility to VNS, but this relationship is likewise intricate and influenced by various factors, and therefore requires further investigation to be understood. Furthermore, no significant gender differences were observed in this study, although the impact of taVNS on SAI function tended to be smaller in females. Given that there are reports from animal studies indicating that VNS has larger effects in females, possibly due to the influence of estrogen on muscarinic acetylcholine in the central nervous system^12,54^, a more extensive investigation with a larger sample size is warranted.

For the limitation of this study, we purposefully did not investigate SICI in order to carefully examine the intervention effects of taVNS on SAI while eliminating confounding factors. However, it is evident that SICI can also contribute to decreases in MEP amplitudes^26,29^, so it will be necessary to thoroughly examine how SICI changes with the enhancement of SAI by taVNS in the future. Additionally, it is essential to investigate whether taVNS activates the noradrenergic system in conjunction with modulating the cholinergic system^4–7,55^. Furthermore, the participants of this study were all young and healthy adults, which may limit the generalizability of our findings to broader populations. Finally, for estimation of the efficacy of taVNS on cholinergic system activation in clinical therapy, after effect of taVNS, i.e., how long the SAI enhancement observed at 15 min after the end of taVNS, as identified in this study, lasts and after how many minutes the SAI returns to baseline, needs to be investigated. Ferthermore, the study design should incorporate pharmacology to better understand the effects of taVNS on cholinergic neural circuits.

In conclusion, in this study of non-invasive vagus nerve stimulation in healthy individuals, we demonstrated that continuous taVNS at 25 Hz enhances the SAI through activation of the cholinergic system.

## Methods

### Participants

Twenty-four healthy adults (12 males and females, mean age ± SD = 24.0 ± 6.1 years) participated in this study. None of the participants were undergoing medical treatment for any condition, and all participants were non-smokers. Twenty-one participants were right-handed with Edinburgh Handedness Inventory scores of 70 or above^56^, and the three other individuals had scores of 50, 6, and − 22, respectively. All participants provided written informed consent before the experiment, which was conducted in accordance with the principles of the Declaration of Helsinki. This study was approved by the ePthics committee of Hiroshima University (No. C-2023-0007).

### Design and procedure

The experiment was conducted in a quiet shielded room with the ambient temperature maintained between 24 and 26 °C. Subjects were seated in a comfortable reclining armchair with a headrest. Forearm immobilization was achieved at 30–45 degrees of supination using a cushion and secured with a right strap. Subjects received either intermittent, continuous, or sham taVNS in a counterbalanced order. To prevent carryover effects, all subjects participated in three experimental sessions on separate days with at least a two-day gap between sessions. The timing for each subject’s participation remained consistent. Participants were instructed to abstain from consuming caffeine for two hours before the experiment. During the initial session, somatosensory evoked potentials (SEP) were recorded to measure the latency of the N20 component. In all sessions, SAI and electrocardiography (ECG) were evaluated before, immediately after, and 15 min after taVNS.

### Transcutaneous auricular vagus nerve stimulation (taVNS)

In this study, we administered stimulation using the tVNS® R stimulator and ear electrodes (tVNS Technologies GmbH, Reichenschwand, Germany). The two circulars iridium-coated titanium surface electrodes were 2 mm in diameter, and conductivity was further enhanced using conductive gel. We specifically selected the left cymba conchae, which is innervated by the sensory auricular branch of the vagal nerve (ABVN), for tVNS. For the sham stimulation, we targeted the earlobe^8–11,14^. The stimulation device was controlled via the dedicated tVNS® Research app (tVNS Technologies GmbH, Reichenschwand, Germany) installed on a smartphone. We employed square-shaped pseudobiphasic pulses with a frequency of 25 Hz, a pulse width of 250 μs, and an intensity set at the midpoint between the tactile and pain thresholds^8,20^. Initial stimulation commenced at 1 mA and the electrode position was gradually adjusted by administrators until participants reported the strongest sensation. Subsequently, the intensity was increased in 0.1 mA increments until participants reported a pain sensation (pain threshold). The stimulus intensity was then reduced in 0.1 mA steps until the sensation disappeared. Finally, the intensity was set at 0.5 mA below the tactile threshold and then increased in 0.1 mA steps until participants reported sensation. This process was repeated until the points of appearance and disappearance of sensation matched (tactile threshold). Although the electrodes and attachments were designed to snugly fit the cymba conchae and earlobe, most participants needed elastic tape to secure them firmly to the auricle skin to ensure consistent current delivery. The duration of intermittent stimulation (28 s on, 32 s off) was 30 min and continuous stimulation lasted 15 min. Sham stimulation of the earlobe was performed for 30 min using intermittent stimulation for twelve of the participants, and 15 min using continuous stimulation for the other twelve participants.

### Somatosensory evoked potentials (SEPs)

We employed a preamplification system (BA1008, Nihon Santeku, Osaka, Japan) to record electroencephalography signals with a bandpass filter of 5–1000 Hz and a sampling rate of 5000 Hz, 200 responses were averaged. A recording electrode was placed 2 cm posterior to C3 (C3’) of the International 10–20 system, with a reference electrode placed on the right earlobe. The impedance of the electrode was kept below 5 kΩ. The bar-type electrode (length: 55 mm; width: 15 mm; electrode distance: 20 mm) was positioned over the right median nerve at the wrist with the cathode positioned proximally. Brief electrical stimulation (0.2 ms) was delivered at a frequency of 3.3 Hz (DS3: Digitimer, Letchworth Garden City, UK). The stimulus intensity was consistently set at approximately 1.2 times the motor threshold.

### Motor evoked potentials (MEPs)

MEPs were recorded from the right abductor pollicis brevis (APB) muscle following transcranial magnetic stimulation (TMS). TMS was conducted using a standard double (figure-of-eight) 70-mm coil connected to a monophasic Magstim 200 stimulator (Magstim Company, Whitland, UK). The coil was positioned tangentially to the scalp with the handle oriented posterolaterally at a 45-degree angle from the midline. We identified the optimal position for activating the right APB muscle by adjusting the coil’s placement within the presumed hand motor area in the left M1 (approximately 4–6 cm lateral and 2 cm anterior of the vertex) using a Brainsight neuronavigation system (Rogue Research, Montreal, Canada) equipped with a Polaris Vicra position sensor system. The motor hotspot was marked as the site where TMS of slightly suprathreshold intensity consistently produced the largest MEP in the APB muscle. The stimulator’s output was adjusted to elicit a thumb movement with an amplitude of 0.8–1.2 mV peak-to-peak (10% of M max) in the relaxed APB muscle. Surface EMG was recorded using disposable silver–silver chloride surface electrodes (1.0 cm diameter) (Blue Sensor, METS, Tokyo, Japan), with recording and reference electrodes positioned over the muscle and tendon. The ground electrode was affixed to the right wrist, positioned between the stimulation electrode and the EMG electrode, utilizing a disposable gel electrode. EMG signals were amplified (100 times) and bandpass filtered (5–500 Hz) through a preamplification system (DL-140; 4 Assist, Tokyo, Japan) and digitized at 10 kHz (PowerLab; AD Instruments, Sydney, Australia). Data were recorded and stored for offline analysis on a personal computer.

### Short-latency afferent inhibition (SAI)

Before TMS, a suprathreshold brief electrical stimulus (200 μs) was delivered to the median nerve at the wrist through bipolar surface electrodes, with the cathode proximal to the anode. Median nerve stimulation was set just above the visible motor threshold to evoke an M-wave with an approximate peak-to-peak amplitude of 0.5–1 mV. Inter-stimulus intervals were synchronized with the individual N20 latency in the SEP plus 2 ms^25^. In randomized order, 15 unconditioned stimuli (TMS stimulation alone) and conditioned stimuli (TMS preceded by peripheral nerve stimulation) were administered at each inter-stimulus interval, with an inter-trial interval of 5 s.

### Electrocardiogram (ECG)

We recorded electrocardiograms (ECG; Maxell, Tokyo, Japan) as the impact of continuous and intermittent taVNS on autonomic nervous system function. Three Ag/AgCl surface electrodes (Blue Sensor, METS, Tokyo, Japan) were placed on the left and right clavicles and costal margins. ECG signals were amplified (1100 times) and bandpass filtered (0.5–20 Hz) through a preamplification system and digitized at 250 Hz. The device used was a specially designed prototype that calculated heart rate (HR) and performed low-frequency (LF)/high-frequency (HF) analysis automatically. Power spectral analysis was performed by calculating areas under the LF (0.04–0.15 Hz) band, a component that reflects sympathetic activity including parasympathetic activity, and the HF (0.15–0.40 Hz) band, which reflects parasympathetic activity. The LF/HF ratio was then calculated to determine the balance of autonomic activity. Higher LF/HF values indicate dominant sympathetic activity, while lower values indicate dominant parasympathetic activity^57^. Accordingly, the ECG device that we used lacked data logging functionality and was not capable of calculating heart rate variance (HVR). ECGs were recorded for 3 min before, immediately after, and 15 min after each administration of taVNS.

### Data and statistical analysis

The latency of the SEP component’s N20 was calculated automatically using EEG analysis computer software (MaP1200A, Nihon Santeku, Osaka, Japan). Likewise, peak-to-peak amplitudes of MEPs were automatically measured by computer software (Scope; AD Instruments, Sydney, Australia). To assess SAI function, the average amplitude of the conditioned MEPs was expressed as a percentage of the average amplitude of the unconditioned MEPs (%SAI: [conditioned MEPs—unconditioned MEPs] / unconditioned MEPs). Heart rate (HR) and the LF/HF ratio were also analyzed.

All data were presented as mean ± standard error of the mean (SEM). Statistical analysis was conducted using SPSS Statistics software version 21 (SPSS; IBM Corp., NY, United States). The normality of the data was assessed using the Shapiro–Wilk test and non-parametric statistical tests were employed if the data did not follow a normal distribution. Two-way repeated measures analysis of variance (ANOVA) was used to analyze the amplitude of unconditioned and conditioned MEPs, %SAI, intensity of taVNS (active and sham stimulation and tactile and pain thresholds), HR, and LF/HF, with the taVNS parameters (intermittent vs. continuous vs. sham) and time (before vs. immediately after vs. 15 min after taVNS) as factors. The sphericity of the data was assessed with Mauchly’s test, and Greenhouse–Geisser corrected significance values were applied when sphericity was violated. Post hoc analysis was performed using Bonferroni’s correction for multiple comparisons. Furthermore, the Pearson correlation coefficient was utilized to assess the relationship between baseline %SAI and its change (Δ%SAI) after taVNS. A single measure of the intraclass correlation coefficient (ICC) (1, 3) was employed to assess the reliability of %SAI before taVNS under three conditions (intermittent, continuous, and sham taVNS). Additionally, differences in Δ%SAI between males and females were assessed using the Mann–Whitney U test. A significance level of 5% was considered statistically significant for all analyses, with results accepted as significant when *p* < 0.05.

## Data Availability

All the data of the current study are available from the corresponding authors on reasonable request.
